# Pre-pandemic SARS-CoV-2-specific IFN-γ and antibody responses were low in Ugandan samples and significantly reduced in HIV-positive specimens

**DOI:** 10.3389/fimmu.2023.1148877

**Published:** 2023-04-19

**Authors:** Hellen Nantambi, Jackson Sembera, Violet Ankunda, Ivan Ssali, Arthur Watelo Kalyebi, Gerald Kevin Oluka, Laban Kato, Bahemuka Ubaldo, Freddie Kibengo, Joseph Ssebwana Katende, Ben Gombe, Claire Baine, Geoffrey Odoch, Susan Mugaba, Obondo James Sande, The COVID-19 Immunoprofiling Team, Pontiano Kaleebu, Jennifer Serwanga

**Affiliations:** ^1^ Medical Research Council (MRC), Uganda Virus Research Institute (UVRI) and London School of Hygiene and Tropical Medicine (LSHTM), Uganda Research Unit, Entebbe, Uganda; ^2^ Department of Immunology, Uganda Virus Research Institute, Entebbe, Uganda; ^3^ Department of Immunology and Molecular Biology, College of Health Sciences, Makerere University, Kampala, Uganda

**Keywords:** SARS-CoV-2, Common-cold coronaviruses, sero-crossreactivity, Pre-existing IFN-γ, Sub-Saharan Africa, Disease severity, Ugandan population, Cross protection

## Abstract

**Introduction:**

We investigated whether prior SARS-CoV-2-specific IFN-γ and antibody responses in Ugandan COVID-19 pre-pandemic specimens aligned to this population's low disease severity.

**Methods:**

We used nucleoprotein (N), spike (S), NTD, RBD, envelope, membrane, SD1/2-directed IFN-γ ELISpots, and an S- and N-IgG antibody ELISA to screen for SARS-CoV-2-specific cross-reactivity.

**Results:**

HCoV-OC43-, HCoV-229E-, and SARS-CoV-2-specific IFN-γ occurred in 23, 15, and 17 of 104 specimens, respectively. Cross-reactive IgG was more common against the nucleoprotein (7/110, 15.5%; p = 0.0016, Fishers' Exact) than the spike (3/110, 2.72%). Specimens lacking anti-HuCoV antibodies had higher rates of pre-epidemic SARS-CoV-2-specific IFN-γ cross-reactivity (p-value = 0.00001, Fishers’ exact test), suggesting that exposure to additional factors not examined here might play a role. SARS-CoV-2-specific cross-reactive antibodies were significantly less common in HIV-positive specimens (p=0.017; Fishers' Exact test). Correlations between SARS-CoV-2- and HuCoV-specific IFN-γ responses were consistently weak in both HIV negative and positive specimens.

**Discussion:**

These findings support the existence of pre-epidemic SARS-CoV-2-specific cellular and humoral cross-reactivity in this population. The data do not establish that these virus-specific IFN-γ and antibody responses are entirely specific to SARS-CoV-2. Inability of the antibodies to neutralise SARS-CoV-2 implies that prior exposure did not result in immunity. Correlations between SARS-CoV-2 and HuCoV-specific responses were consistently weak, suggesting that additional variables likely contributed to the pre-epidemic cross-reactivity patterns. The data suggests that surveillance efforts based on the nucleoprotein might overestimate the exposure to SARS-CoV-2 compared to inclusion of additional targets, like the spike protein. This study, while limited in scope, suggests that HIV-positive people are less likely than HIV-negative people to produce protective antibodies against SARS-CoV-2.

## Introduction

Since the onset of severe acute respiratory syndrome (SARS-CoV-2) outbreak in Uganda and other Sub-Saharan African countries, COVID-19 disease severity and mortality have been lower than in Europe, Asia, and the Americas (1, 2). Several hypotheses were proposed to explain this discrepancy, including cross-reactive adaptive immune responses to SARS-CoV-2 and antigenically related human coronaviruses (3) that cause common seasonal colds. Studies assessing antibody prevalence to SARS-CoV-2 in pre-pandemic serum specimens observed a significant increase in the prevalence of cross-reactivity among sera in SSA compared to other continents (4). Antigenic cross-reactivity between SARS-CoV-2 and human coronaviruses such as HCoV-229E, HCoV-NL63, HCoV-HKU1 and HCoV-OC43 has been demonstrated in other geographical regions through the detection of cross-reactive humoral and cellular immune responses (5). Different conserved human coronaviruses (HCoVs) cross-reactive B-cell epitopes against SARS-CoV-2 N protein are detected in a significant fraction of individuals not exposed to this pandemic virus (6). While the spike and nucleocapsid are the most immunogenic (7), the nucleocapsid is more conserved across various human coronaviruses; thus, cross-reactivity can be expected. The less conserved spike protein is more susceptible to mutations, especially in the receptor binding domain (RBD); thus, cross-reactivity to it would be a potential protective correlate. The cross-reactive antibodies of interest are IgG, as these are the hallmarks of B-cell memory (8).

Pre-COVID-19 pandemic plasmas from Tanzania and Zambia were shown to have a higher prevalence of serological cross-reactivity to SARS-CoV-2, HCoV-HKU1, HCoV-OC43, HCoV-NL63 and HCoV-229E, compared to specimens from the USA (4). A robust pre-existing humoral immunity against SARS-CoV-2 is thus entirely plausible as a reason for the observed lower disease severity and mortality. Although this can be attributed to the presence of pre-existing cross-reactive binding antibodies, it remains to be seen if, and to what capacity and breadth, such antibodies can neutralise SARS-CoV-2 or have other mechanisms of conferring protection (9, 10).

Cross-reactive immune responses to SARS-CoV-2 have been shown in pre-pandemic cohorts and proposed to contribute to host protection. Pre-existing T-cell-mediated immunity to SARS-CoV-2, from prior exposure to antigenically related seasonal coronaviruses, has been demonstrated in other populations previously unexposed to SARS-CoV-2 (11). Pre-existing T-cell contribution to asymptomatic or mild disease, rapid viral clearance, and differences in seroconversion have been suggested (12). Efforts continue to understand the potential role of T-cell memory induced by prior exposure to seasonal coronaviruses in reducing COVID-19 disease severity, and findings have remained conflicting. Epitopes specific to seasonal coronaviruses and those cross-reactive to SARS-CoV-2 have been defined, and their potential roles in vaccine design and pathogenesis have been postulated (13). Airway-resident SARS-CoV-2- T cell cross-reactivity correlated with the magnitude of human seasonal coronavirus immunity highlighting the potential to harness cross-reactive T cells in vaccine design (14). Higher frequencies of non-spike T cell cross-reactivity in PCR-negative exposed household contacts underscored the importance of cross-reactive T cell memory in protecting SARS-CoV-2-naïve contacts from infection (15). In contrast, a case-control study found no association between baseline human coronavirus cross-reactive antibodies and protection against SARS-CoV-2 infection but suggested that pre-existing human coronavirus immunity might aggravate SARS-CoV-2 infection instead, which is critical in considering emerging variants (16). Detection of a pre-existing cellular and humoral immunity against SARS-CoV-2 and other coronaviruses in each population is significant, mainly because the occurrence is not ubiquitous in all populations (17).

For almost three years since the emergence of COVID-19, Uganda, like many sub-Saharan African countries, has experienced lower COVID-19 and a less severe epidemic than other regions worldwide. Prior cross-reactivity is widely postulated as a possible explanatory feature for the observed differences and is essential for interpreting diagnostic strategies. Consequently, we used pre-COVID-19 Ugandan specimens to examine the presence, prevalence, and magnitude of pre-existing SARS-CoV-2-specific cross-reactive immune responses to test the hypothesis that pre-pandemic natural immunity acquired by some human populations may have contributed to the epidemic outcome.

## Materials and methods

### Study design and population

This retrospective study estimated the levels of pre-COVID-19 pandemic cross-reactivity to SARS-COV-2 using 110 specimens collected between January 2009 and February 2015 for future optimisation of immunological assays. Of 110 available specimens, 68 (68.12% were HIV-negative, and 36 (32.72%) were HIV-1 positive. 35 of the 36 HIV-1 positive were on cotrimoxazole prophylaxis due to HIV-related progressed CD4 counts, 6 lacked HIV-1 serostatus information, and 2 lacked ELISpot data. Ten of the 36 HIV positive subjects had CD4 count data available, with a median of 483.0 (IQR 231.58-678.2 CD4 cells/ml).

Correspondingly, 110 plasmas and 104 PBMC specimens were available to screen for pre-epidemic anti-SARS-CoV-2 cross-reactive IgG and IFN-γ responses, respectively. Specimens with no documented HIV status were excluded in analyses by HIV stratification. All participants provided written informed consent for their specimens’ storage and future use. Study ethical approval was granted by the Uganda Virus Research Institute (UVRI) Research and Ethics Committee (IRB Ref: GC/127/233) and the Uganda National Council for Science and Technology (Ref: HS1030).

### SARS-CoV-2, HCoV-229E and HCoV-OC43 peptide pools

The SARS-CoV-2 and the seasonal human coronavirus OC43 (HCoV-OC43) and 229E (HCoV-229E) peptides arrays were 12-17 mers long, overlapping by ten amino acids, obtained from the BEI Resources Repository, NIAID, USA, selected based on availability. The SARS-CoV-2 array constituted 181 spike peptides (S; GenPept: QHO60594 NR-52402), 59 nucleoprotein peptides (N; GenPept: QHO60601, NR-52404), 31 membrane peptides (M; GenPept: QHO60597, NR-52403) and ten envelope peptides (E; NR-52405). Single peptides were combined based on the SARS-CoV-2 proteome region into fourteen spike (S) pools. The S1 region comprised all SARS-CoV-2 spike peptides spanning amino acid 13-684 (NTD: 13-305, RBD: 331-528, and OTHERS: 1-12, 306-330, 529-684). The S2 region comprised all spike peptides spanning amino acids 685-1211 (18). The S1 region was subdivided into NTD-1, NTD-2, NTD-3, RBD-1, and RBD-2 pools, while all other S1 peptides were arranged in ascending order and divided into OTHERS 1 and OTHERS 2. The S2 region was divided into seven peptide pools, namely S2-1, S2-2, S2-3, S2-4, S2-5, S2-6 and S2-7. The nucleoprotein (N) was divided into two pools (N1: N1-N30 and N2: N31-N59), while membrane (M) and envelope (E) had one pool each, summarised in **Tables 1** and **2**. Common human coronavirus peptides included 226 S protein peptides combined into one pool (NR-3011, BEI Resources) and 195 individual 229E S protein peptides combined into a single pool (NR-3010, BEI Resources). All peptide pools were reconstituted and used at a final concentration of 2μg/ml per peptide.

**Table 1:** Pooling arrangement of peptides from BEI Resources.

	Region	Number of Overlapping peptides to Pool	Peptide Numbers from the List	Website links
SARS-CoV-2 Peptides	M	31	1-31	https://www.beiresources.org/Catalog/BEIPeptidesandPeptideArrays/NR-52403.aspx
	E	10	1-10	https://www.beiresources.org/Catalog/BEIPeptidesandPeptideArrays/NR-52402.aspx
	N1	30	1-30	https://www.beiresources.org/Catalog/BEIPeptidesandPeptideArrays/NR-52404.aspx
	N2	29	31-59	https://www.beiresources.org/Catalog/BEIPeptidesandPeptideArrays/NR-52404.aspx
Common Cold Peptides	OC43	226	1-226	https://www.beiresources.org/Catalog/BEIPeptidesandPeptideArrays/NR-53728.aspx
	229E	195	1-195	https://www.beiresources.org/productinformationsheet/tabid/784/default.aspx?doc=80254.pdf

**Table 2:** Pooling arrangement SARS-CoV-2 spike peptides from JPT.

	Region	Number of Overlapping peptides pooled	Peptide Numbers from the List	SARS-CoV-2 spike amino acids
SARS-CoV-2 Peptides	NTD 1	24	4-27	13-305
	NTD 2	24	28-51	
	NTD 3	23	52-74	
	RBD 1	24	83-106	331-528
	RBD 2	24	107-130	
	OTHERS 1	21	1, 77-80, 133-148	1-12, 306-330 (SD1/2), 529-684 (SD1/2)
	OTHERS 2	21	149-169	
	S2 1	19	172-190	685-1211
	S2 2	19	191-209	
	S2 3	19	210-228	
	S2 4	19	229-247	
	S2 5	19	248-266	
	S2 6	19	267-285	
	S2 7	15	286-300	

### Detection of T-Cell responses using an IFN-γ ELISpot assay

The cryopreserved cells were permitted to rest in growth media at a temperature of 37°C overnight. Subsequently, the median percentage (interquartile range) of cell recovery was recorded, with values of 78% (74-84) on day one and 63% (45.75-75) on day two. Moreover, the corresponding percentage viability values were determined, with recorded values of 99% (interquartile range 98.25-99) and 100% (interquartile range 99-100) on days one and two, respectively. These findings indicate the successful recovery and viability of the cryopreserved cells following the resting period, highlighting their potential suitability for subsequent experimental procedures.

Duplicate wells pre-coated with anti-human IFN-γ capture antibody were seeded with 100,000 PBMCs/well and incubated with respective peptide pools at 37°C for 18 hours in a 5% CO_2_-in-air environment. Captured IFN-γ was detected with a biotinylated anti-human IFN-γ detection antibody and horseradish peroxidase-conjugated streptavidin, developed using a Vector Novared kit (Vector Laboratories, SK-4800, CA, USA), as described by the manufacturer. Control wells included donor specimens of known reactivity to SARS-CoV-2, HCoV-229E and HCoV-OC43 spike and phytohemagglutinin (PHA) treated PBMCs. Background reactivity was computed from four wells containing cells with mock (media with DMSO peptide diluent) and four with media only. Spots were counted using an AID ELISpot Reader (GMBH, Germany). IFN-γ secreting T-cells were enumerated as Spot-forming units (SFU), and the overall response as SFU per million PBMC (SFU/10^6^ PBMC). The test acceptance criteria were ≥300 spots in PHA wells, ≤ 10 in each of the four wells containing cells plus mock, and ≤ 5 in each of the four media-only wells. Positive responses were deduced after subtracting two times the mean of background wells. Test wells with a net response ≥ 55 SFU/10^6^ PBMC were considered positive.

### Detection of virus-specific binding antibodies using ELISA

SARS-CoV-2-specific S and N binding IgG optical densities (450nm) and concentrations (ng/ml) were quantified using an in-house optimized conventional ELISA, as described before (19). Briefly, 96-well flat-bottomed membrane-binding plates (Greiner Bio-One) coated with 3 μg/ml recombinant S or N protein in PBS were incubated with serum specimens diluted 1:100 in PBS-T containing 1% Bovine Serum Albumin (BSA). A secondary monoclonal horseradish-peroxidase conjugated goat anti-human IgG (IgG-HRP) antibody (catalogue no. A0170, Sigma) was added for 1 h before washing to remove any excess unbound antibody. Tetra-methyl-benzidine (TMB) substrate solution was added for 3 minutes, and the reaction stopped with 2.5% (0.68M) Hydrochloric acid. Bound IgG was quantified as optical densities (ODs) read on an ELx808 microplate reader (BioTek, Winooski, VT, USA) at 450nm. Net OD values were computed by subtracting the OD values of blank wells. Antibody concentrations were extrapolated from serially diluted standards and calibrated to BAU/ml using WHO international standards.

### Preparation of SARS-CoV-2 Pseudoviruses for neutralization assays

SARS-COV-2 pseudoviruses were generated by co-transfection of 293T cells with MLV-CMV-firefly plasmids encoding the firefly luciferase gene, MLV gag-pol and Wuhan-strain SARS-COV-2 spike plasmids, using the PEIMAX (Polysciences) transfection reagent. Culture supernatants containing the pseudovirus particles were clarified of cells using a 0.45µm filter, titrated and stored at -80°C. Working aliquots were quickly thawed at room temperature and diluted to pre-determined working concentrations to give sufficient luciferase signals in infected cells.

### SARS-CoV-2 pseudotyped virus neutralization assay

A pseudovirus neutralization assay was used to determine whether the identified cross-reactive binding antibodies neutralised SARS-COV-2. Prior to testing, sera were heat-inactivated at 56°C for 30 minutes. In order to determine the potential neutralizing effect of the sera, serial dilutions ranging from 1:10 to 1:320 were prepared and incubated with fixed dilutions of the virus for a period of 1 hour. This incubation was carried out at a temperature of 37°C in an environment consisting of 5% CO2 in air. Duplicate wells were utilized to ensure accuracy of the results. Ten thousand 293T cells expressing human ACE2 receptors were added, and the wells were further incubated for 72 hours at 37°C, 5% CO_2_. The wells were then incubated with Promega 1X lysis buffer and Bright-Glo Luciferase reagent for 15min. Luciferase expression in infected cells was measured using a Perkin-Elmer victor X3 luminometer. Neutralization titres were computed from linear interpolating plotted virus infectivity curves as 50% inhibitory titres (IC50). Inhibitory titres were reciprocals of serum dilutions that reduced the relative luminescence in test wells by 50% compared to the virus control wells. The lower detection limit of the SARS-CoV-2 neutralisation assay was ten, so virus titres less than ten were deemed undetectable.

### Statistical analysis

GraphPad Prism 9.4.1 (GraphPad Software Inc, San Diego, California, USA) and R Version 4.1 were used for the statistical analyses and graphical data representation; p-values ≤ 0.05 were considered statistically significant. A two-tailed Mann-Whitney test was used to compare median IFN-γ responses to different peptide pools and median nucleocapsid and spike-directed antibody levels. P-values *≤* 0.05 were considered statistically significant.

## Results

### Pre-pandemic IFN-γ cross-reactivity to SARS-CoV-2 and human coronavirus structural proteins was detected at a low frequency, but primarily in HIV-1 negatives

**Figure 1:**
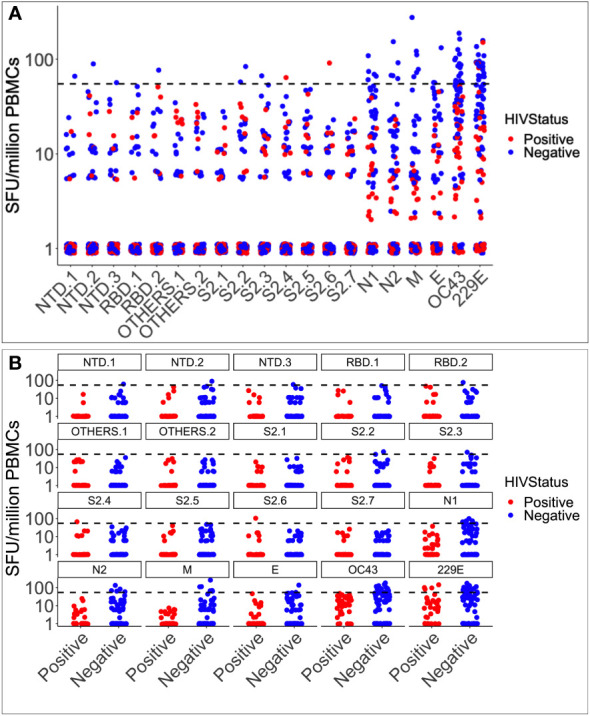
Pre-epidemic IFN-γ responses to SAR-CoV-2, HCoV-229E and HCoV-OC43. Illustrates the virus-specific IFN-γ responses to 18 SARS-CoV-2 and two human coronavirus peptide pools (HuCoVOC43 and HuCoV229E). IFN-γ response magnitudes are depicted and quantified as spot-forming units per million PBMC cells. The horizontal dotted red line represents the cut-off threshold for a positive response of 55 SFU/million PBMCs **(A)**. The data represents 104 pre-COVID specimens classified as positive in red and negative in blue based on HIV serostatus **(B)**.

Pre-epidemic IFN-γ cross-reactivity to SARS-CoV-2 was assessed in 104 specimens, 36 of which were HIV-1 positive and 68 were HIV negative. Cross-reactivity with HCoV-OC43, HCoV-229E, and SARS-CoV-2 occurred in 23 (22.1%), 15 (14.4%), and 17 (16.4%) of the 104 specimens, respectively. Cross-reactivity IFN-γ frequencies were greater to the human coronaviruses (29; 27.9%) than to SARS-CoV-2 (17; 16.3%), although the difference was not statistically significant (Fishers’ Exact test, p-value = 0.065; **Figure 1A**). Rates of cross-reactivity did not differ between the two human coronaviruses HCoV-OC43 (15; 14.4%) and HCoV-229E (23; 22.1%). SARS-CoV-2-specific IFN-γ cross-reactivity of was greater in HIV-negative specimens (15/68; 22.06%) than in positives (2/36; 5.56%); Fishers’ Exact test, p-value 0.0484, **Figure 1B**. HuOC43 cross-reactivity was also more prevalent in HIV-negative specimens (15 of 68) than in HIV-positive specimens (0 of 36); p = 0.0011. Cross-reactivity to HuCoV229E did not substantially vary by HIV status (p = 0.08). When examined based on the most often targeted SARS-CoV-2 antigens for populational surveillance, the frequencies of nucleoprotein (8 of 104) and spike-directed (5 of 104) IFN-γ cross reactivity did not vary. Similarly, anti-spike IFN-γ cross-reactivity did not vary between HIV-negative (3 of 68) and HIV-positive (2 of 36) tissues; Fishers’ Exact test p-value > 0.05. These findings show that pre-existing immunity to SARS-CoV-2 occurred in Uganda, most likely due to prior exposure to other coronaviruses, and that SARS-CoV-2 antigens were not targeted more frequently than other coronavirus antigens. Cross-reactive IFN-γ responses to SARS-CoV-2 antigens were substantially more prevalent in HIV-negative specimens, than in HIV-positive specimens suggesting that the lower level of pre-existing immunity reported in Ugandans may be partly explained by HIV-positive persons’ diminished capacity to generate a significant IFN-γ response to SARS-CoV-2 antigens. Thus, the Ugandan data gives an insightful view into the pre-existing immunity to SARS-CoV-2 in various populations and the variables that might impact it.

### Pre-pandemic anti-SARS-CoV-2 IFN-γ cross-reactivity was uncommon and comparable across the nucleoprotein and spike, but it was broader and more frequent in the HIV-1 negatives

**Figure 2:**
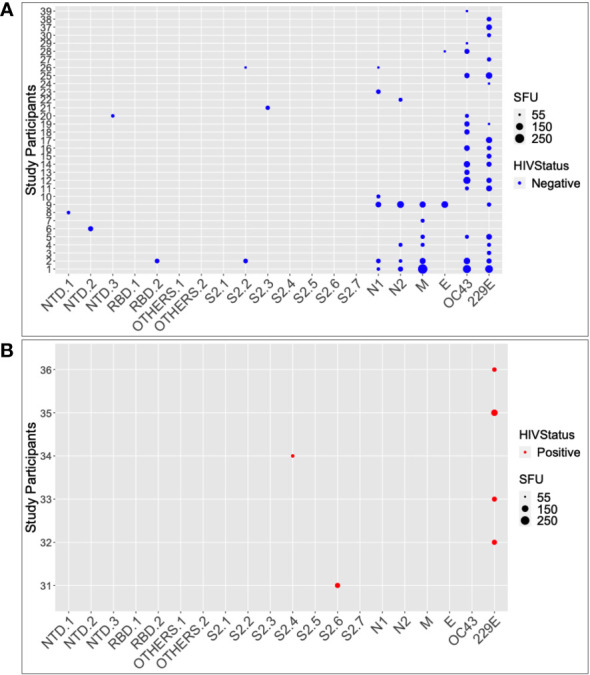
The SARS-CoV-2 and HuCoV peptide pools targeted by HIV-1 status. Summarizes the individual responses to each peptide pool for 39 of the participants who responded positively to at least one peptide pool. The data is stratified by HIV serostatus, with HIV negatives in blue **(A)** and HIV positives in red **(B)**. The larger the size of the circle, the greater the response.

Six of the seven pooled SARS-CoV-2 antigenic regions tested positive for IFN-γ. The evaluated regions comprised the nucleoprotein, spike, NTD, RBD, envelope, membrane, and SD1/2 (categorised here as “OTHERS”). Seventeen of the 104 pre-pandemic specimens tested positive for SARS-CoV-2-specific IFN-γ cross-reactivity; eight specimens (7.69%) targeted the nucleoprotein; eight (7.69%) targeted the spike; six (5.76%) targeted the membrane; two (1.92%) targeted the envelope; and no cross-reactivity occurred to “OTHERS”, (**Figure 2**). In total, 16.3% of the specimens were positive for pre-epidemic IFN-γ cross-reactivity with SARS-CoV-2, with the nucleoprotein and the spike being the most frequently recognized targets. Cross-reactive antigenic pools were more common in HIV-negative specimens (6 of 7 pools) than in HIV-positive specimens (1 of 7 pools); Fishers’ Exact test, p-value = 0.029 (**Figures 2A, B**).

Among the 15 HIV-negative SARS-CoV-2 responders, the number of SARS-CoV-2 cross-reactive pools ranged from 1 to 5, with a median of 1 (IQR; 1-2 pools). Only two of the 36 HIV-positive specimens tested positive for SARS-CoV-2-specific IFN-γ cross-reactivity, and both were against the S2 region of the SARS-CoV-2 spike protein. Using the Wilcoxon test, the median IFN-γ levels in HIV-negative participants were significantly greater than those of HIV-positive participants (p = 0.00038; 15; IQR 5–20 SFU/million PBMCs vs. 10; IQR 6.25–35 SFU/million PBMCs). Taken together, these data demonstrate the presence of pre-pandemic cross-reactive IFN-γ in this population, implying that the immune system had been primed to respond to SARS-CoV-2 antigens prior to the outbreak. These data also show that HIV-positive individuals are less likely to produce cross-reactive IFN-γ against SARS-CoV-2 than those who are HIV-negative. These findings have significant implications for the development of SARS-CoV-2 cross-protective vaccines, which may need to consider HIV infection status when assessing immunogenicity and efficacy.

### Cross-reactive IFN-γ responses to pre-pandemic SARS-CoV-2 and human coronaviruses were weakly correlated

**Figure 3:**
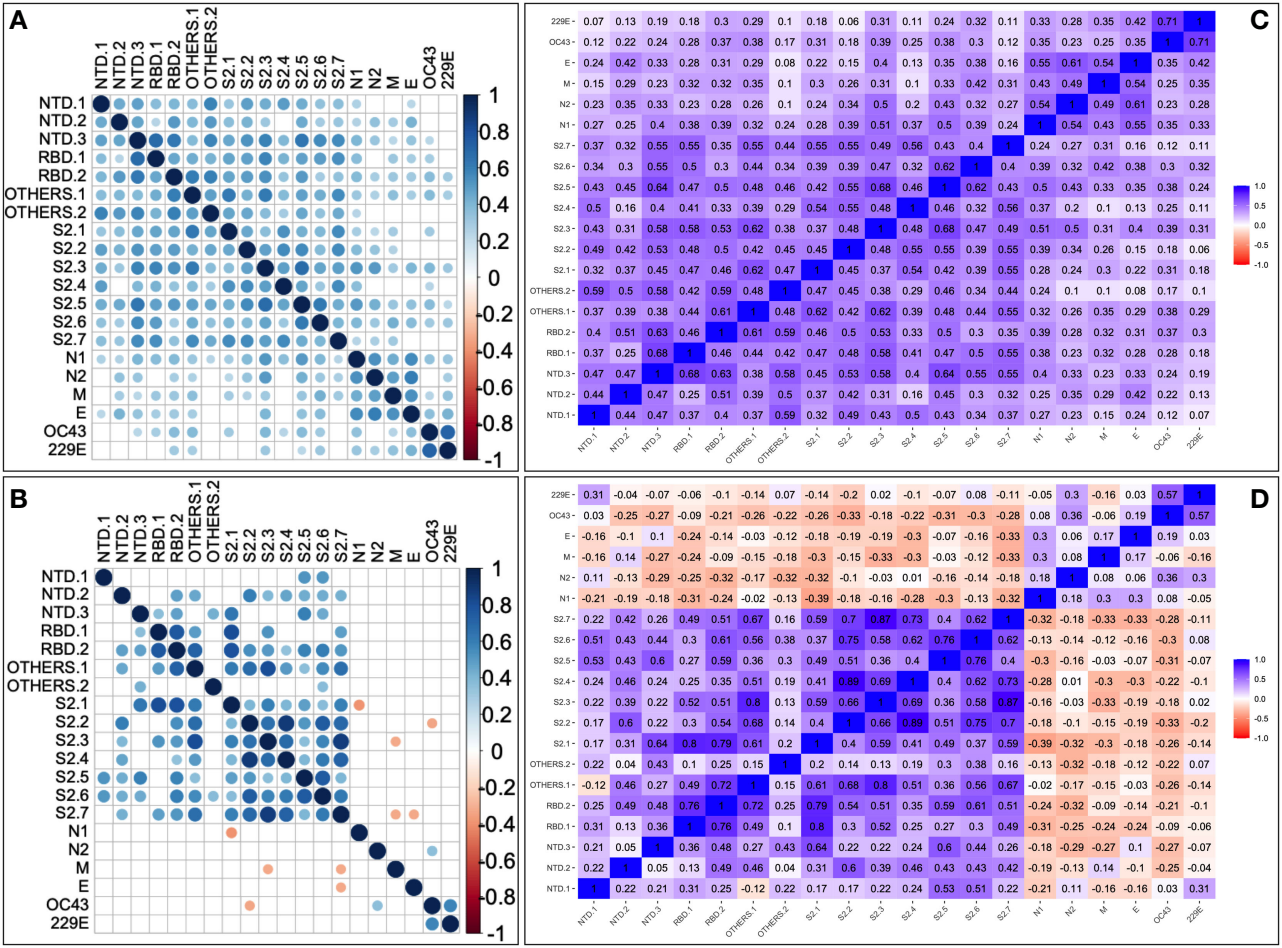
Pairwise correlation between SARS-CoV-2 and HuCoV-directed IFN-γ. Depicts correlation plots of all examined SARS-CoV-2 and human coronavirus peptide pools for 68 HIV-negative **(A)** and 36 HIV-positive **(B)** specimens using Spearman's rank correlation test. Blue dots represent a positive correlation, while red dots represent a negative correlation. The darker the circle, the stronger the correlation, and the lighter the circle, the weaker the correlation. The correlation matrices show correlation coefficients between peptides in HIV-positive **(C)** and HIV-negative **(D)** specimens using Spearman's rank correlation. P-values of 0.05 or less are considered significant. Non-significant correlations are indicated by blank cells.

Next, correlations between historical human coronavirus IFN-γ cross-reactivity and the pre-epidemic SARS-CoV-2-specific IFN-γ cross-reactivity were explored. Anti-HuCoV IgG-deficient specimens (10/10, or 100%) had a higher frequency of cross-reactive SARS-CoV-2-specific IFN-γ responses than anti-HuCoV IgG-expressing specimens (7/29, or 24.1%); Fishers’ exact test, p-value = 0.00001. Specifically, pre-epidemic SARS-CoV-2-specific cross-reactive IFN-γ responses were more common in the absence of anti-HuCoV antibodies, suggesting that exposure to additional variables not assessed here may have contributed to this phenomenon. Pairwise peptide comparisons for the correlation between SARS-CoV-2 and HuCoV-specific IFN-γ responses in both HIV-negative (**Figure 3A**) and positive (**Figure 3B**) specimens consistently revealed weak correlations. Regardless of HIV serostatus, robust positive correlations occurred between cross-reactive IFN-γ specific to each of the two human coronaviruses, HCoV-OC43 and HCoV-229E (**Figures 3C, D**).

The data showed the presence of pre-epidemic SARS-CoV-2-specific T-cell cross-reactivity in this cohort, these could not be fully explained by prior T cell cross-reactivity to human coronaviruses. It implies that most of the epidemic SARS-CoV-2 cross-reactive T cells were specific to SARS-CoV-2, rather than derived from prior coronavirus exposure. The findings suggest that prior coronavirus exposure may have had little to no impact on the pre-epidemic SARS-CoV-2-specific T cell pool, meaning that these T cells detected during the epidemic were likely generated in response to the emergence of the SARS-CoV-2 epidemic itself. Taken together, these findings agree with the existence of pre-existing HuCoV IFN-γ cross-reactivity, but they do not entirely explain the prevalence of SARS-CoV-2 IFN-γ responses before the epidemic.

### Pre-epidemic SARS-CoV-2-directed IgG cross-reactivity was more common with the nucleoprotein than the spike, but only in HIV-negative individuals

**Figure 4:**
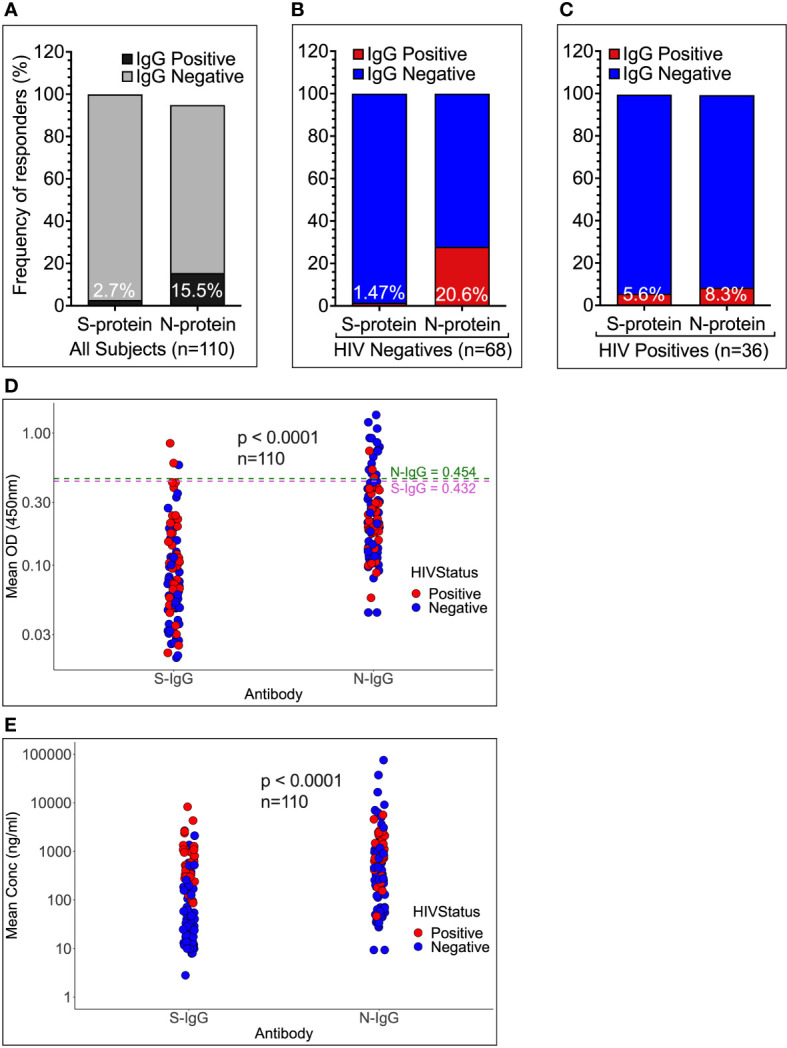
Frequencies and magnitudes of SARS-CoV-2 -specific cross-reactive IgG binding antibodies by HIV status. The frequency of pre-epidemic cross-reactive anti-SARS-CoV-2 binding IgG antibodies in Ugandan pre-pandemic sera is depicted in **Figure 4**. 110 specimens **(A)** were stratified by HIV-1 negative **(B)** and positive serostatus **(C)**, and the proportions of subjects with cross-reactive IgG binding antibodies against the SARS-CoV-2 spike and nucleoprotein illustrated. **(D, E)** summarises the medians of means of duplicate IgG antibody OD values in nm and concentration levels in ng/ml, respectively. The cut-off OD values are shown by the dashed lines.

Cross-reactive IgG against the nucleoprotein was more frequent (17/110, 15.5%) than against the spike (3/110, 2.72%), p = 0.0016, Fishers’ Exact test (**Figure 4A**). Cross-reactive IgG was still significantly higher against the nucleoprotein (14/68, 20.59%) than the spike (1/68, 1.47%) in HIV-negative individuals (Fishers’ Exact test, p = 0.017, **Figure 4B**), but not in HIV-positive individuals (Nucleoprotein: 3/36, 8.33% vs. Spike: 2/37, 5.55%), **Figure 4C**. Nucleoprotein-directed IgG antibody optical densities (0.211; IQR 0.134 - 0.364nm) and concentrations (529.75; IQR 180.78-1154.33 ng/ml equivalent to 265.3; IQR 90.55 – 578.18 BAU/ml) were also significantly higher against the nucleoprotein than the spike (optical density 0.072 nm; IQR 0.048-0.120 and concentration 95; IQR 24.45-483.20 ng/ml equivalent to 37.83; 10.22 – 201.89 BAU/ml), Wilcoxon matched-pairs signed rank test, all p-values of 0.0001). (See **Figures 4D, E**). Taken together, these findings suggest that the nucleoprotein, rather than the spike protein, is a more important target for developing SARS-CoV-2-directed cross-reactive antibodies in HIV-negative individuals. Antibodies to the nucleoprotein were less specific to SARS-CoV-2 and were much more likely to cross-react with viruses from other families. As a result, surveillance efforts focusing on the nucleoprotein may overestimate SARS-CoV-2 exposure compared to other targets, such as the spike protein, while underestimating exposure, particularly in populations with a higher prevalence of HIV-positive individuals. When calculating and interpreting SARS-CoV-2 exposure for sero-epidemiological surveillance, HIV serostatus is a crucial factor to consider.

### Pre-epidemic SARS-CoV-2-directed IgG cross-reactive antibodies were not neutralizing and did not correlate with IFN-γ cross-reactivity

Finally, the functionality of the detected spike-directed IgG cross-reactive antibodies was determined using the pseudotyped virus neutralisation assay.

**Figure 5:**
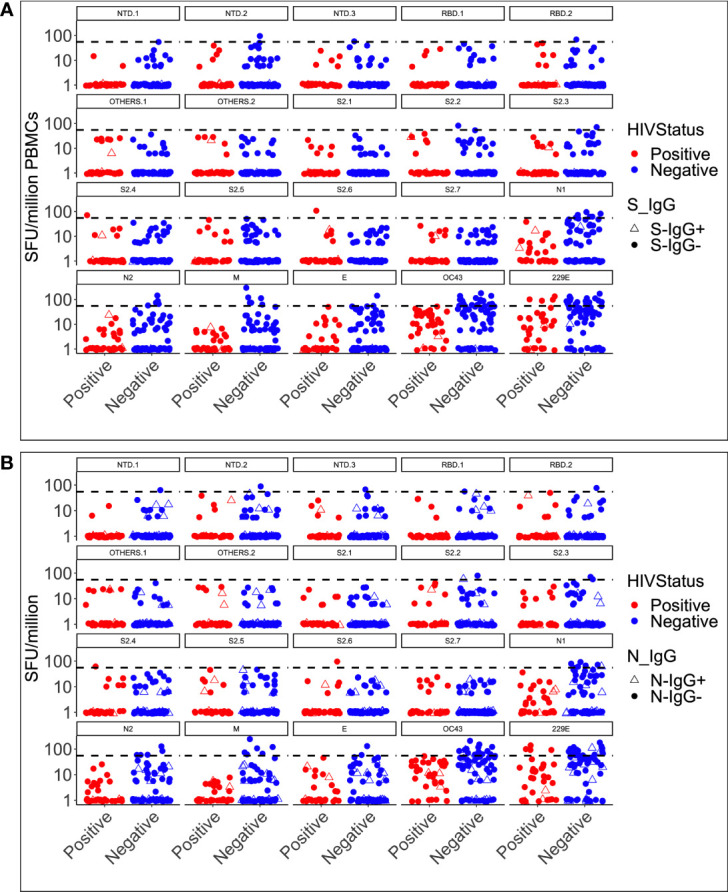
IFN-γ cross-reactivity categorised HIV status and spike **(A)** and nucleoprotein **(B)** IgG response. Depicts the simultaneous induction of SARS-CoV-2-specific IgG antibodies and IFN-γ cross-reactive responses in 104 specimens with antibody and T-cell data against the spike **(A)** and the nucleoprotein **(B)**. The data is presented according to HIV serostatus, with HIV-positive individuals in red and HIV-negative individuals in blue. Open triangles represent participants with cross-reactive antibodies and IFN-γ responses.

Based on the SARS-CoV-2 neutralisation assay, the lowest dilution used was ten, rendering virus titres below ten undetectable. All responses obtained from the assay were found to be below the positivity detection limit. Consequently, it can be inferred that the pre-epidemic anti-spike SARS-CoV-2 antibodies were incapable of neutralising the wild-type SARS-CoV-2 pseudovirus *in vitro* (**Table 3**). Individuals who were infected with other coronaviruses prior to the pandemic, but not with SARS-CoV-2, may not have sufficient immunity to protect against the currently circulating strains of SARS-CoV-2. The inability of pre-pandemic antibodies to neutralize SARS-CoV-2 indicates that prior exposure did not result in SARS-CoV-2 immunity prior to the pandemic; thus, any immunity against the virus at the time of the pandemic was most likely the result of novel immune responses elicited by natural exposure to SARS-CoV-2 or its vaccine. The findings highlight the importance of continuing research into SARS-CoV-2 immunity mechanisms to identify protective antibodies and develop effective treatments and vaccines. Cross-reactive IFN-γ against SARS-CoV-2 or human coronaviruses was not induced in any of the three specimens with cross-reactive SARS-CoV-2 spike-IgG antibodies (**Figure 5A**). Despite the presence of cross-reactive SARS-CoV-2 IgG antibodies, most specimens lacked an IFN-γ response. Only one of the 17 specimens with nucleoprotein-directed cross-reactive IgG antibodies produced virus-cross-reactive IFN-γ to SARS-CoV-2, while three produced virus-cross-reactive IFN-γ to either of the two human coronaviruses (**Figure 5B**). According to these findings, existing spike-directed antibody cross-reactivity may not be specific to SARS-CoV-2. The lack of an IFN-γ response in the presence of SARS-CoV-2 antibodies suggests that SARS-CoV-2-directed IgG antibodies did not elicit a novel IFN-γ response in response to natural SARS-CoV-2 or vaccine exposure. Our understanding of the immune system’s ability to respond to SARS-CoV-2 is still limited.

**Table 3:** SARS-CoV-2 directed IgG cross-reactive neutralizing antibody titres. Summarises the responses of nine participants tested for neutralising antibodies against SARSCoV-2.

	LogNT90	NT90	LogNT50	NT50
PARTICIPANTS	1.954	1	1.699	1
	1.954	1	1.699	1
	1.954	1	1.699	1
	1.954	1	1.699	1
	1.954	1	1.699	1
	1.954	1	1.699	1
	1.954	1	1.699	1
	1.954	1	1.699	1
	1.954	1	1.699	1

The highest antibody titre that neutralised 50% and 90% of the virus, NT50 and NT90, respectively, were recorded. The antibody titres were logarithmically scaled to account for minimal differences. Notably, the lower detection limit of the SARS-CoV-2 neutralisation assay was 10, rendering virus titres below this threshold undetectable. Consequently, all responses obtained from the assay fell below the detection limit for positivity. For statistical analysis, NT50 values below det5ection were assumed to be 1.699, while undetectable NT90 values were equated to 1.954.

## Discussion

This study used pre-pandemic Ugandan specimens to examine the levels of pre-existing cross-reactive cellular and humoral responses to seasonal human coronaviruses and how they fit with Uganda’s lower COVID-19 disease severity. We found that pre-pandemic IFN-γ T-cell responses were detectable against the structural proteins of SARS-CoV-2 and HCoV-OC43 and HCoV-229E human coronaviruses. The SARS-CoV-2-specific IFN-γ cross-reactivity targeted the nucleoprotein, spike, membrane, and envelope viral proteins and was broader and stronger in HIV-1-negative specimens than in HIV-1-positive ones. The correlation between pre-pandemic SARS-CoV-2-directed IFN-γ cross-reactivity and human coronavirus-directed IFN-γ cross-reactivity was weak, indicating that cross-reactive T-cells to seasonal human coronaviruses do not fully explain the detection of pre-epidemic SARS-CoV-2-specific T-cell cross-reactivity. SARS-CoV-2-specific IgG antibody cross-reactivity was detectable, but it was primarily directed against the nucleocapsid protein. Cross-reactive, spike-directed antibodies did not neutralize SARS-CoV-2 *in vitro*, and no correlation occurred between antibody and IFN-γ responses.

Various hypotheses have been suggested linking the comparatively lower SARS-CoV-2 pathogenesis infection rates in sub-Saharan Africa to possible cross-protection due to prior exposure to seasonal human coronaviruses. The pre-pandemic serological cross-recognition of human coronaviruses was shown to be more prevalent in SSA than in USA specimens (4). Our findings agree with the prevalent serological cross-reactivity to SARS-CoV-2 in pre-epidemic Ugandan specimens reported by others before (20). However, we observed a much lower serological prevalence of 15.5% of 110 specimens (HCoV-OC43 and HCoV-229E combined) compared to the 87.5% reported by Mulabbi et al. (20). Seroprevalence of spike-directed cross-reactivity, the hallmark of antibody protection was even much lower occurring at a frequency of 2.72% of 110 pre-pandemic specimens. Differences in screening methodology could explain the much lower prevalence in the same population. Mulabbi et al.’s study (20) screened with a commercial ELISA kit using wells pre-coated with a nucleoprotein, aa 340-390 peptide; the test specimens were not diluted, and the cut-off threshold was deduced by adding 0.15 to the mean of negative wells; conditions which in our hands would have resulted in the majority of the specimens being positive. Here, we used a validated in-house ELISA that uses the complete nucleoprotein proteome, with all test specimens first prediluted 100-fold to define a virus-specific response. The assay limits of detection and cut-off thresholds used were established and validated for the Ugandan population (19). The simultaneous ELISpot screening of our specimens using seven SARS CoV-2 antigenic regions to establish a comparable human coronavirus T-cell cross-reactivity prevalence of 27.9% corroborates our antibody prevalence data.

We have established that the cross-reactive binding antibodies detected here are not specific or functional against the prototype SARS-CoV-2 viruses. We have also shown that pre-existing T-cell cross-reactivity is comparable to that reported in South Africa, where a dual colour ELISpot assay detected IFN-γ responses in 29.9% and IL-2 in 39.2% with a combined response rate of 51.6% (21). Higher levels of cross-reactive T-cell frequencies of up to 50% reported in a U.S. study are likely attributable to evaluations of a broader range of seasonal coronavirus strains and a broader range of viral epitopes (11). The broader selection of strains and epitopes likely triggered a higher frequency of cross-reactive T-cells in the U.S. In general, the methodology of these other studies differed slightly from ours in terms of lower cut-off values for positive IFN-γ responsiveness (20 SFU/10 6 PBMCs) and the use of flow cytometry to detect additional T-cell responses (55 SFU/106 PBMCs).Nevertheless, even with these differences in the methodologies, the results of both our study and others showed that some people had some level of pre-existing cross-reactive T cells prior to being exposed to SARS-CoV-2.

We observed a trend toward decreased cross-reactivity in HIV-1-negative individuals of median CD4 counts of 483.0, IQR: 231.5 - 678.2 CD4 cells/μl. HIV-1 seropositive specimens were obtained from patients receiving Cotrimoxazole prophylaxis, which is routinely administered to patients with low CD4+ T-lymphocyte counts in order to prevent potentially fatal bacterial infections. Immunocompromised status may have had a further impact on the frequency of detectable cross-reactivity, as other studies have demonstrated that HIV-1-infected individuals have lower cross-reactive responses to SARS-CoV-2 (4, 22), particularly those with lower CD4 counts (23).

Nevertheless, these results demonstrate that exposure to HCoV-229E and HCoV-O43 has been endemic in Uganda, indicating the potential for the presence of pre-existing cross-reactive immune responses to SARS-CoV-2. Their low prevalence and the lack of correlation between SARS-CoV-2 and human coronavirus-specific immunity suggest that pre-existing cross-reactive immunity may not significantly account for the comparatively mild impact of COVID-19 in Uganda. The data highlight the importance of further studies to understand the role of pre-existing immune responses to HCoV-229E and HCoV-O43 in protecting against SARS-CoV-2 infection in Uganda. Others showed that low comparative T-cell epitope homology between SARS-CoV-2 and common cold coronaviruses was deemed insufficient to offer cross-protective cellular responses in other contexts as well and may therefore not be a critical correlate of low disease severity (24). Here, the cross-reactive IgG antibody response was stronger against the nucleocapsid than the spike, showing that the observed cross-reactive antibody response targets the most conserved regions of coronaviruses (25, 26).

This research had some limitations. While cross-reactive antibodies lacked neutralizing activity, other protective antibody functions not evaluated here are mediated by non-evaluated antibody-dependent effector mechanisms, such as antibody-dependent cellular cytotoxicity and Fc effector functions (4, 18, 27). As a result, we recommend additional research into the diverse effector mechanisms of these cross-reactive antibodies, as they may provide valuable insights into the development of a pan-coronavirus-directed vaccine. By elucidating the molecular and cellular mechanisms through which cross-reactive antibodies function, this research could provide a framework for the development of universal vaccines against coronaviruses, providing an invaluable contribution to the global health landscape. For determining the relationship between prior cross-reactivity and immunity, a longitudinal study design with pre- and post-epidemic specimens is optimal. Such a study design would compare pre-existing levels of cross-reactivity to post-epidemic levels of immunity to determine the extent to which prior cross-reactivity may be responsible for protection against a novel virus. We are conducting a follow-up study to investigate the association between pre-existing cross-reactivity and disease outcome. Further, we recommend investigating the variability in genetic influence on the production of such cross-reactive antibodies, which was not determined in this study, and exploring their epitope specificities to inform vaccine design targets. In addition, due to reagent accessibility constraints, we only tested two human coronavirus strains. To better understand the entire spectrum of prior cross-reactivity, more seasonal coronavirus strains and viral epitopes will need to be evaluated. In addition, it would be instructive to know what the results would have been with a much larger, more heterogeneous, and more representative sample, as this study was limited to only 110 participants, 36 of whom were HIV-seropositive.

Also, only spike-based PNA and nucleocapsid cross-binding antibodies were studied. Functional non-neutralizing antibody responses to SARS-CoV-2 were not evaluated. Because cross-reacting, binding antibodies may have effector mechanisms other than neutralization, evaluating the efficacy of other effector functions, such as N-directed functionality, is critical for understanding the full spectrum of immunological protection that a specific antibody may provide (28, 29). Antibodies, for example, can be evaluated for their ability to activate complement-dependent cytotoxicity (CDC), antibody-dependent cellular phagocytosis (ADCP), and antibody-dependent cell-mediated cytotoxicity (ADCM) (ADCC). Finally, live viral assays shed light on how antibodies interact with viruses and the effector functions that they can perform in addition to spike-based PNA functionality. To fully understand the effector mechanisms of cross-reacting binding antibodies, it is critical to use live viral assays in conjunction with spike-based PNA and nucleocapsid tests.

In conclusion, these results confirm the presence of pre-existing SARS-CoV-2 specific cross-reactivity, but do not demonstrate that these antibodies are entirely specific to SARS-CoV-2. More research is needed to fully understand the presence and clinical significance of pre-existing SARS-CoV-2 specific cross-reactivity. The findings shed new light on the potential for the nucleoprotein to induce cross-reactive immunity against SARS-CoV-2 and suggest that surveillance efforts targeting the nucleoprotein may overestimate the exposure to SARS-CoV-2 relative to other targets, like the spike protein. Specifically, the data underscores the need for multiple serologic assays targeting a variety of SARS-CoV-2 antigens to more accurately measure exposure. In addition, the data revealed a lower degree of cross-reactivity in HIV-positive individuals compared to healthy individuals, indicating that pre-existing SARS-CoV-2 specific cross-reactive immunity may not be a characteristic shared by all populations. Preexisting cross-reactivity must be considered when developing an accurate serological test for SARS-CoV-2 infection, as this study demonstrates.

## Data availability statement

The raw data supporting the conclusions of this article will be made available by the authors, without undue reservation.

## Ethics statement

The studies involving human participants were reviewed and approved, and ethical approval granted by the Uganda Virus Research Institute Research and Ethics Committee (IRG Ref: GC/127/233) and the Uganda National Council for Science and Technology (Ref: HS1030). The participants provided written informed consent to participate in this study.

## Author contributions

Conceptualized the work and drafted the initial manuscript: JSer, HN, VA and JSem. Clinical management and collection of specimens: BU, and FK. Conducted the laboratory assays: HN, JSem, LK, JSK, BG, SM, CB, GO, GKO, IS, AWK and “The COVID-19 Immunoprofiling Team”. Data analysis: JSer, VA, HN, JSem, GKO and LK. Scientific Oversight and Supervision: JSer, OJS, SM and PK. All authors wrote and reviewed the manuscript. Reviewed and authorized the final manuscript. JS and PK. All authors contributed to the article and approved the submitted version.

## COVID-19 Immunoprofiling Team

Opio Solomon; Ejou Peter; Namuyanja Angella, Hermilia Christine Akoli.
